# Mixed Adenosquamous Cell Carcinoma of the Prostate with Paired Sequencing on the Primary and Liver Metastasis

**DOI:** 10.3390/curroncol31050178

**Published:** 2024-04-24

**Authors:** Emmanuella Oyogoa, Maya Sonpatki, Brian T. Brinkerhoff, Nicole Andeen, Haley Meyer, Christopher Ryan, Alexandra O. Sokolova

**Affiliations:** 1 Department of Medicine, Oregon Health and Science University, Portland, OR 97239, USA; 2 Department of Microbiology, Oregon State University, Corvallis, OR 97331, USA; 3 Department of Pathology and Laboratory Medicine, Oregon Health and Science University, Portland, OR 97239, USA; 4 Department of Internal Medicine, Mayo Clinic, Rochester, MN 55902, USA; 5 Division of Hematology and Medical Oncology, Knight Cancer Institute, Oregon Health and Sciences University, Portland, OR 97239, USA

**Keywords:** squamous cell carcinoma of the prostate, mixed adenosquamous cell carcinoma of the prostate, prostate cancer, prostate-specific membrane antigen positron emission tomography (PSMA PET)

## Abstract

This report aims to shed light on the intricate challenges encountered during the diagnosis and treatment of an uncommon variant of prostate cancer—mixed adenosquamous cell carcinoma of the prostate. Prostate cancers of this nature pose distinctive diagnostic and therapeutic dilemmas due to their rarity and complex histological composition. We present a case of a 63-year-old man with metastatic prostate cancer, featuring adenocarcinoma with squamous cell differentiation, who underwent a multimodal treatment approach. The patient responded to first-line carboplatin, docetaxel, and androgen deprivation therapy, followed by androgen receptor pathway inhibitor (ARPI) maintenance. However, disease progression led to radiation therapy and a subsequent switch to Lutetium (177Lu) vipivotide tetraxetan after chemotherapy challenges. Comprehensive genetic profiling revealed shared mutations in the prostate and liver lesions, emphasizing the role of targeted therapies. Prostate-specific membrane antigen (PSMA)-targeted therapy resulted in a notable PSA decline. This case highlights the evolving treatment landscape for rare prostate cancers, integrating genetic insights for tailored interventions. In conclusion, squamous cell carcinoma (SCC) of the prostate is rare, emphasizing the imperative for enhanced comprehension in diagnosis and management. Our case suggests the potential efficacy of ARPI and PSMA-targeted therapies. Our findings advocate for a more nuanced approach to the management of this rare prostate cancer variant, leveraging genomic insights for personalized treatment strategies. This exploration serves as a foundation for further research and clinical considerations in addressing the challenges posed by mixed adenosquamous cell carcinoma of the prostate.

## 1. Introduction

Prostate cancer stands as the most prevalent non-cutaneous malignancy among American men [1], with an estimated 288,300 new cases in 2023 [2]. Adenocarcinoma constitutes 95% of prostate cancer cases, representing the predominant histology. Conversely, squamous cell carcinoma (SCC) of the prostate is a rare manifestation accounting for less than 1% of cases [3,4]. Typically, mixed adenosquamous cell carcinoma of the prostate emerges as a transformation of adenocarcinoma, while in certain cases, it is thought to be primary, although the origin of primary prostate SCC is still debated [5]. The genetic and molecular intricacies of prostate SCC remain inadequately explored [5]. This case report presents a case of mixed adenosquamous cell carcinoma of the prostate, providing paired sequencing data from both the primary prostate tumor and liver metastasis.

## 2. Case Report

**Figure 1:**
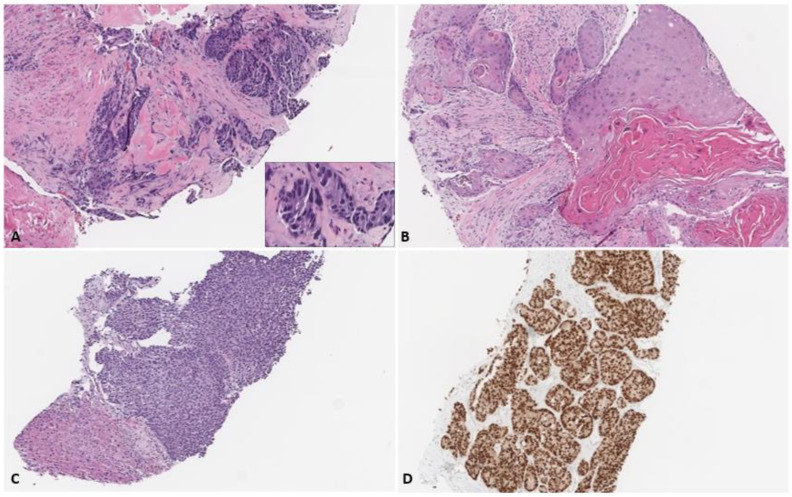
Biopsies of the (**A**) prostate showed high-grade adenocarcinoma (inset showing high power view of glandular component (400×)) with areas of (**B**) keratinizing squamous differentiation. Liver biopsies showed a (**C**) poorly differentiated adenocarcinoma with (**D**) immunoreactivity for the prostate marker NKX3.1 (all images at 100×).

A 63-year-old man with past medical history of diabetes type 2, a family history of prostate cancer in his father and no smoking history was diagnosed with metastatic prostate cancer at the age of 61 after presenting with urinary retention. At the time of presentation, the PSA was 14.8 ng/mL. A prostate biopsy revealed prostate adenocarcinoma with squamous cell differentiation and a Gleason score of 5 + 4 (Figure 1A,B). A bone scan showed osseous lesions in the ribs and pelvic bones, and CT of the chest, abdomen and pelvis revealed visceral metastasis to the liver. The stage at diagnosis was T3AN1M1c. A CT-guided liver biopsy revealed poorly differentiated carcinoma, positive for NKX3.1 via immunohistochemistry (Figure 1C).

Comprehensive genetic profiling, using institutional tumor next-generation sequencing panel Genetrails, of the pretreatment prostate biopsy showed genomic alterations in *PIK3CA* p.E542K, *MUTYH* p.G393D and androgen receptor (AR) amplification (11 copies). Other potential clinically significant mutations include the following: *ATRX* loss, *MLH1* loss, * PTEN* loss, *SUFU* loss, *TP53* K123R and loss, *SMAD4* loss and *CDKN1B* splice site. Genomic studies from the liver lesion biopsy obtained before the initiation of therapy showed the same mutations as those from the prostate, including *AR* amplification (17 copies), *PIK3CA* p.E542K and *MUTYH* p.G393D. Germline testing revealed monoallelic *MUTYH* p.G396D alteration and was negative for *TP53* and DNA damage repair gene alterations.

**Figure 2:**
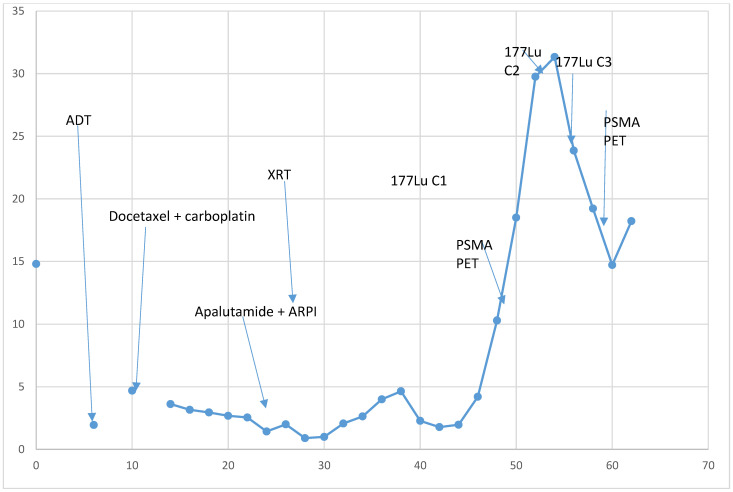
PSA levels and corresponding treatment course with time on the X axis and PSA (ng/mL) on the Y axis.

**Figure 3:**
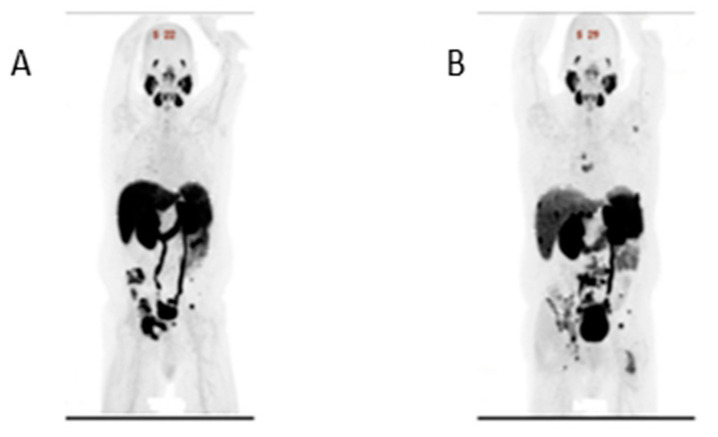
PSMA PET imaging. (**A**) PSMA imaging obtained after completion of chemotherapy treatment with hepatic, pelvic bone and pelvic lymph node uptake. (**B**) PSMA imaging obtained 6 months after (**A**) while on ADT and ARPI. Compared to 3A, increased osseous lesion, hepatic lesions and lymph nodes. New uptake present in spinal bone. Decreased uptake in pelvic bone.

The initiation of carboplatin and docetaxel, in conjunction with androgen deprivation therapy (ADT), was prompted by the SCC component identified in the patient’s prostate biopsy. He responded well to therapy with PSA decline from 14 ng/mL to 2 ng/mL and radiographic response (decrease in size and extent of hepatic metastases and lymphadenopathy and stable osseous lesions with no new sites of metastases). After completing six cycles of carboplatin and docetaxel, he continued ADT and started AR pathway inhibitor (ARPI) therapy due to his adenocarcinoma component. He started two ARPIs, apalutamide and abiraterone, on a clinical trial (Figure 2) [6]. PSA further declined during ARPI therapy, with a PSA nadir of 0.9 ng/mL. After 25 weeks of therapy, his PSA started rising to 4 ng/mL. His restaging imaging showed interval development of a single new lytic destruction and soft tissue component of the right posterior acetabular metastasis, no other new disease, decreased hepatic metastases and no change in the enlarged abdominal/pelvic lymph nodes. Lutetium-177 therapy was discussed and PSMA PET imaging was performed, which showed PSMA uptake in all known metastases: liver, lymph nodes and bone metastasis (Figure 3A). The patient opted for radiation therapy (XRT) to the single lesion’s progression, the acetabulum, and a continuation of ARPI and ADT therapy. PSA declined reaching a nadir of 1.78 ng/mL. Four months after XRT, PSA started rising again during ARPI therapy, and imaging showed a new liver metastasis and increases in the size of the soft tissue component of osseous metastasis. The patient had a repeat PSMA PET scan (Figure 3B) and opted to undergo Lutetium (177Lu) vipivotide tetraxetan therapy. Unfortunately, this therapy became unavailable due to supply issues, and the patient received one cycle of cabazitaxel and carboplatin chemotherapy before initiating PSMA-targeted therapy. Initially, the patent showed a response to Lutetium (177Lu) vipivotide tetraxetan therapy with a PSA decline from 33 ng/mL to 15 ng/mL after the first cycle. Unfortunately, PSA started rising after cycle 3, and he had radiographic progression after cycle 3 with new and enlarging pulmonary, hepatic and bone metastasis. The patient was hospitalized for altered mental status, raising concerns about leptomeningeal metastasis despite the absence of clear radiographic evidence. Although the altered mental status resolved, the patient later developed pneumonia. Opting for a transition to comfort care, he passed away.

## 3. Discussion

Outcome data for SCC of the prostate are limited but suggest a more aggressive phenotype and worse treatment outcomes compared to adenocarcinoma [7]. For localized SCC cases, several reports have suggested the efficacy of multimodal therapy [8]: a combination of radiation and/or radical prostatectomy with androgen deprivation therapy (ADT) and chemotherapy [9]. There is no standard of care for metastatic SCC, and chemotherapy is typically offered as the first-line therapy along with ADT [10]. Generally, the same treatment options are available for both SCC and adenocarcinoma. However, chemotherapy doublets are preferred in SCC of the prostate. The treatment response to systemic therapies for squamous cell carcinoma is less than what is observed for adenocarcinoma [11].

SCC of the prostate is associated with a more aggressive disease phenotype compared to adenocarcinoma, and is more likely to have metastasized to bone, liver or lungs at the time of diagnosis [12], to have lower prostate-specific antigen (PSA) with advanced disease [13] and has worse survival [14]. In 11 case reports of primary squamous cell carcinoma of the prostate, the median overall survival was 11 months with survival ranging between 3 months and 6 years [3,15,16,17,18,19,20,21,22,23,24]. At the time of diagnosis, PSA is often low, even in the setting of metastases. It is often challenging to identify the origin of SCC, and some experts propose that SCC originates from the urothelium and then migrates to the prostate [5,11,13,25]. The presence of prostate-specific alterations, such as *TMPRSS2-ERG* or *SPOP,* can help determine the prostate primary origin of the SCC [25]. The data on the somatic mutation profile of the SCC of prostate cancer is limited.

In this case, we present matching pretreatment biopsies of prostate and liver metastasis. Overall genomic fundings were very similar in these two biopsies and showed activation of the PI3K/AKT pathway, *AR* amplification and *T53* mutation.

In the presented case, PI3K/AKT pathway activation was evident with *PIK3CA* p.E542K alteration and *PTEN* loss. PI3K/AKT pathway activation is common in many cancer types, including SCC of different origins (cervix, oral cavity, head and neck and skin) [26]. It is present in about 40% of early prostate cancer cases and up to 70–100% of advanced cases [27,28,29,30]. It is unclear if PI3K/AKT activation in the presented case is associated with the SCC or adenocarcinoma component. Two other prostate SCC case reports reported *PTEN* alterations; one case reported *PTEN* alteration in both primary and metastatic biopsies [25], suggesting it might be an early alteration in the tumorigenesis of SCC of the prostate. PI3K/AKT pathway activation is associated with resistance to androgen deprivation therapy and poor outcomes in conventional adenocarcinoma of the prostate. Several therapeutic strategies are being evaluated to target the PIK/AKT pathway, with AKT inhibitors being the most promising. Phase III trial, IPATential150, demonstrated improved radiographic progression-free survival with the AKT inhibitor Ipatasertib in combination with abiraterone compared to placebo plus abiraterone among patients with mCRPC with PTEN-loss [31]. Another phase III trial evaluating the role of capivasertib, an AKT inhibitor, is ongoing [32]. Clinical trial participation with an AKT inhibitor was a potential therapeutic option for our patient but was not available when Lu-117 therapy started.

*AR* amplification is a common alteration in castration-resistant adenocarcinoma of the prostate and is present in up to 50% of cases [33,34,35,36]. However, it is rarely present in untreated prostate tumors [37]. In this case, of SCC of the prostate, *AR* amplification was present in both the liver biopsy and the prostate tissue that were obtained prior to the initiation of treatment, suggesting an early event. *AR* amplification is associated with resistance to the *AR* pathway inhibitors [38,39]. The presented case had *AR* amplification and SCC component of the tumor—both predictive of poor response to ARPI. However, the patient had a longer-than-expected response to ARPI and completed 10 months of ARPI therapy with a stable disease, except single oligometastatic progression treated with XRT. This case suggests that there could be a role for ARPI therapy in mixed adenosquamous cell carcinoma prostate tumors.

As is found in many cancers, we detected a somatic mutation of *TP53* in the primary tumor and liver metastases, and there was no evidence of a germline alteration on genetic testing. Somatic *TP53* mutations are common in many tumors, including prostate tumors. Sweeney et al. found that about 46% (n = 37/76) of patients who had no prior treatment with abiraterone or enzalutamide had a somatic *TP53* mutation. In comparison, 41% (n = 108/262) of patients treated with abiraterone and/or enzalutamide had a somatic *TP53* mutation [40]. Although somatic *TP53* mutation did not appear to play a major role in the characterization of SCC of the prostate in this case, germline *TP53* mutations were recently shown to have an association with increased risk of developing prostate cancer [41].

PSMA is a mostly prostate-specific transmembrane protein with 100- to 1000-fold higher expression in prostatic adenocarcinoma compared to benign prostate [42]. Up to 87% of patients with mCRPC have PSMA-avid tumors [43]. PSMA uptake among the SCC of the prostate has not been characterized. In the presented case, all known metastases had PSMA uptake, and the patient pursued PSMA-directed therapy with the initial response, which unfortunately was not durable. This case highlights the potential role of PSMA PET and PSMA-directed therapy (e.g., Lutetium (177Lu) vipivotide tetraxetan) in the SCC of the prostate.

Overall, SCC of the prostate is a rare disease. This case highlights the need for a better understanding of the diagnosis, treatment and management of pure and mixed adenosquamous cell carcinoma of the prostate. Tumor sequencing can help to identify the origin of SCC and suggest therapeutic approaches. Our case suggests a role for ARPI and PSMA-targeted therapies in mixed adenosquamous cell carcinoma of the prostate. Future directives should include the role of targeted therapy and screening for certain somatic and germline mutations present in prostate cancer. In particular, more research and clinical trials are needed for targeted treatment in pure and mixed adenosquamous cell carcinoma of the prostate.

## Data Availability

No new data were created or analyzed in this study. Data sharing is not applicable to this article.
